# Bunched, the *Drosophila *homolog of the mammalian tumor suppressor TSC-22, promotes cellular growth

**DOI:** 10.1186/1471-213X-8-10

**Published:** 2008-01-28

**Authors:** Silvia Gluderer, Sean Oldham, Felix Rintelen, Andrea Sulzer, Corina Schütt, Xiaodong Wu, Laurel A Raftery, Ernst Hafen, Hugo Stocker

**Affiliations:** 1 Institute of Molecular Systems Biology, ETH Zürich, Wolfgang-Pauli-Str. 16, 8093 Zürich, Switzerland; 2 Zoological Institute, University of Zürich, Winterthurerstr. 190, 8057 Zürich, Switzerland; 3 The Genetics Company Inc., Wagistr. 27, 8952 Schlieren, Switzerland; 4 Cutaneous Biology Research Center, Massachusetts General Hospital/Harvard Med. School, Charlestown, MA 02129, USA; 5 Burnham Institute for Medical Research, 10901 North Torrey Pines Road, La Jolla, CA 92037, USA; 6 PFC Pharma Focus AG, Chriesbaumstr. 2, 8604 Volketswil, Switzerland

## Abstract

**Background:**

Transforming Growth Factor-β1 stimulated clone-22 (TSC-22) is assumed to act as a negative growth regulator and tumor suppressor. TSC-22 belongs to a family of putative transcription factors encoded by four distinct loci in mammals. Possible redundancy among the members of the TSC-22/Dip/Bun protein family complicates a genetic analysis. In *Drosophila*, all proteins homologous to the TSC-22/Dip/Bun family members are derived from a single locus called *bunched *(*bun*).

**Results:**

We have identified *bun *in an unbiased genetic screen for growth regulators in *Drosophila*. Rather unexpectedly, *bun *mutations result in a growth deficit. Under standard conditions, only the long protein isoform BunA – but not the short isoforms BunB and BunC – is essential and affects growth. Whereas reducing *bunA *function diminishes cell number and cell size, overexpression of the short isoforms BunB and BunC antagonizes *bunA *function.

**Conclusion:**

Our findings establish a growth-promoting function of *Drosophila *BunA. Since the published studies on mammalian systems have largely neglected the long TSC-22 protein version, we hypothesize that the long TSC-22 protein is a functional homolog of BunA in growth regulation, and that it is antagonized by the short TSC-22 protein.

## Background

Tumorigenesis is frequently associated with a loss of a tumor suppressor, allowing tumor cells to become self-sufficient in growth signals, to become insensitive to growth-inhibitory signals, or to evade apoptosis (reviewed in [1]). Thus, the functional characterization of tumor suppressors is key to a better understanding of the signaling events leading to aberrant growth.

Transforming Growth Factor-β1 stimulated clone-22 (TSC-22) is a putative negative growth regulator and tumor suppressor in mammals. *TSC-22 *has first been isolated as a TGF-β1 responsive gene from a mouse osteoblastic cell line [2]. It encodes a putative transcription factor that binds to DNA *in vitro *via its TSC-box [3]. *TSC-22 *expression has been found to be lowered in different mouse and human tumors, including liver [4], brain [5], prostate [6], and salivary gland tumors [7]. Consistently, downregulation of *TSC-22 *enhances growth in the salivary gland cell line TYS [7], whereas upregulation of *TSC-22 *is associated with apoptosis [8,9] and growth inhibition [10]. Increased *TSC-22 *expression also correlates with growth inhibition in primary human prostatic cancer cells [11,12]. Furthermore, in the mammary carcinoma cell line T47D, *TSC-22 *is a target gene of progesterone, which is used to treat hormone dependent breast tumors [13]. However, *TSC-22 *has also been found to be upregulated in renal cell carcinoma, challenging its proposed function in tumor suppression [14]. Furthermore, most studies on the role of TSC-22 in tumor formation rely on cell culture experiments, and no information is available on the *in vivo *function of TSC-22 in growth regulation.

The genetic characterization of TSC-22 in mammals is hampered in two ways. First, the *TSC-22 *locus gives rise to two transcripts encoding a longer and a shorter isoform (TSC22D1.1 and TSC22D1.2, respectively). They share the C-terminally located TSC-box and a leucine zipper domain, but their N-termini are distinct. In most of the studies the two isoforms were not examined separately, or only the short isoform TSC22D1.2 has been analyzed. The possibility of diverse (or even antagonizing) functions of the TSC-22 isoforms has been largely neglected. Second, there are four genomic loci (*TSC22D1 *to *TSC22D4*) encoding TSC-22/Dip/Bun family members with diverse functions in mammals. All TSC-22/Dip/Bun proteins possess a TSC-box and a leucine zipper. *TSC22D3 *encodes three short isoforms with different N-termini, and a recent study shows that murine TSC22D3 isoforms have differential functions in cultured kidney cells [15]. One isoform, TSC22D3.2 or Gilz (glucocorticoid-induced leucine zipper), has been investigated intensively. Gilz is induced by glucocorticoids, is highly expressed in lymphoid tissue, and plays a role in the regulation of T cell receptor mediated cell death [16-19]. Besides its function in the immune system, Gilz seems to be important for the aldosterone response and sodium homeostasis of cultured kidney cells [20,21]. Via its N-terminus, Gilz binds to NF-kappaB [22], to c-Jun and c-Fos [23], and to Raf-1 [24]. Furthermore, Gilz is a direct FoxO3 target gene [25]. The function of TSC22D2 (TILZ4 = TSC-22 related inducible leucine zipper 4) is less well understood. In humans, two very similar long TSC22D2 isoforms are known [Swiss-Prot:O75157], and mice have several *TSC22D2 *transcripts potentially coding for short TSC22D2 isoforms with distinct N-termini [26]. TSC22D2 is involved in the osmotic stress response of mouse kidney cells [26]. Finally, TSC22D4 (THG-1 = TSC-22 homologous gene-1) can form heterodimers with TSC-22 (TSC22D1.2) [27] and is important in pituitary development in mice [28]. Since the potential redundancy among the various TSC-22/Dip/Bun family members renders a genetic analysis in mammals very difficult, it is important to assess the *in vivo *function of TSC-22 in a simpler model organism.

*Drosophila melanogaster *is a suitable model organism to study growth regulation. For example, the involvement of insulin signaling [29-31] or of the proto-oncogene dMyc [32] in growth control has been genetically analyzed in *Drosophila*. In addition, screens for genes restricting growth have identified the Hippo-Salvador-Warts signaling cassette that may also have a tumor suppressor function in humans [33,34]. The *Drosophila *genome contains a single gene, *bunched (bun)*, that encodes proteins homologous to the TSC-22/Dip/Bun family members. *bun *has been found to influence the development of the embryonic peripheral nervous system [35], to be expressed during eye development [36], and to be required for proper oogenesis [37]. Like *TSC-22/Dip/bun *genes in mammals, the *Drosophila bun *gene gives rise to alternatively spliced transcripts (six different transcripts, *bun-RA *to *bun-RF*), and little is known about the functions of the individual proteins so far.

Here we report that *bun *functions as a positive growth regulator in *Drosophila*. In a tissue-specific screen for genes involved in growth control, we have isolated eight *bun *alleles. We demonstrate that only the long Bun isoform, BunA/F, promotes cellular growth.

## Results

### Identification of *bun *as a positive growth regulator

**Figure 1:**
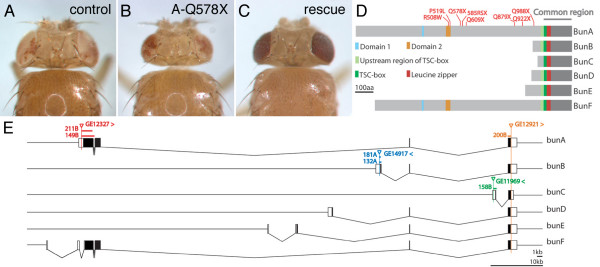
**Identification of *bunA *as positive growth regulator**. Eye-specific reduction of *bunA *function by means of eyFLP/FRT-mediated mitotic recombination results in a reduction of eye and head size (B) as compared to the control (A). This growth deficit is rescued by overexpression of a *bunA *transgene (C). (D) Schematic representation of the six Bun protein isoforms. The putative Bun transcription factors have distinct N-termini but an identical C-terminal region (common region), including the TSC-box (DNA-binding) and an adjacent leucine zipper (homo- and heterodimerization) encoded by the very 3' *bun *exon (E). All Bun isoforms except for BunC also contain a conserved region N-terminally to the TSC-box that is present in all mammalian TSC-22/Dip/Bun family members. In addition, BunA and BunF possess two domains in their N-terminal regions that are conserved among mammalian homologs ([13], domain 1 aligns to BunA amino acids 369-82 [Swissprot:Q24523-1]). The eight EMS-induced mutations isolated in the eyFLP/FRT screen (indicated in red) affect only BunA and BunF. (E) The genomic region of *bun *according to FlyBase [39]. The six *bun *transcripts share the last exon but have distinct 5' exons. UTRs are shown in white and ORFs in black. P-element insertions used for the jump-out screens and deletions obtained in these screens are indicated. Arrowheads indicate the directions of transcription that can be driven by the respective EP insertions. The P-element *GE12327 *and the deletions derived from it as well as the EMS-induced alleles affect both *bunA *and *bunF *but are referred to as *bunA *alleles. Genotypes are: (A) *y, w, eyFLP/y, w; FRT40A, w*^+^, *cl^*2L3*^/FRT40A*^*iso*^; (B) *y, w, eyFLP/y, w; FRT40A, w*^+^, *cl*^2*L*3^*/FRT40A, bun*^*A*-*Q*578*X*^; (C) *y, w, eyFLP/y, w; FRT40A, w*^+^, *cl*^2*L*3^*/FRT40A, bun*^*A*-*Q*578*X*^; *ey-Gal4, GMR-Gal4/UAS-bunA*.

In a tissue-specific genetic screen aiming at the identification of mutations affecting size in *Drosophila *(eyFLP/FRT assay, Methods), we recovered a complementation group consisting of eight EMS-induced recessive lethal alleles that produced a small head (pinhead) phenotype (Figure 1B). Subsequent mapping (Methods) narrowed down the candidate region to the chromosomal interval 33E7-33F2 comprising five candidate genes and 5' exons of the gene *bunched *(*bun*), which had previously been implicated in several developmental processes, namely in embryogenesis [38], neurogenesis [35], eye development [36], and egg shell development [37]. Three lines of evidence indicated that the pinhead complementation group corresponded to the *bun *gene. First, recessive lethal *bun *P-element alleles (*00255*, *04230*, *06903*, and *rI043*) failed to complement the EMS-induced alleles recovered in our mutagenesis. Second, sequencing of the *bun *ORF revealed a point mutation in each of the EMS alleles (Figure 1D). Finally, overexpression of a *bun *transgene rescued, at least partially, the recessive lethality and the pinhead phenotype associated with the alleles recovered in the screen (Figure 1C). Thus, *bun *is the gene responsible for the pinhead phenotype and encodes a protein required to positively regulate growth.

The genomic locus of *bun *spans 90 kb and comprises at least 12 (partially overlapping) exons (Figure 1E). Based on the existence of ESTs, the *bun *locus gives rise to at least six different transcripts (*bun-RA *– *bun-RF*) [39]. Since the *bunD-F *transcripts have been annotated only recently, our study mainly focused on *bunA-C*. *bunA *and *bunF *have largely overlapping ORFs, but the BunF protein lacks the first 109 N-terminal amino acids present in BunA. The six *bun *transcripts have distinct promoter regions and code for six putative transcription factors that contain a DNA-binding domain called TSC-box and an adjacent leucine zipper that likely serves as a dimerization domain (Figure 1D). Proteins of the TSC-22/Dip/Bun family are found in various organisms ranging from *C. elegans *to mammals. Apart from the TSC-box and the leucine zipper, the amino acid sequences of the *Drosophila *Bun proteins are poorly conserved when compared to their mammalian homologs. However, for long TSC-22/Dip/Bun protein isoforms, namely human TSC22D1.1 (TSC-22 long), human TSC22D2.1, human TSC22D4, and *Drosophila *BunA and BunF, two short stretches of high conservation but unknown function have been identified (domain 1 and domain 2, Figure 1D, [27]). Interestingly, two alleles recovered in our screen carry a mutation leading to an amino acid exchange in domain 2, supporting the functional importance of this domain. The other six EMS alleles cause a premature termination of translation.

### The short isoforms BunB and BunC are not involved in growth regulation

Our EMS alleles of *bun *are the first point mutations in the *bun *locus. Strikingly, all eight mutations exclusively affect the long Bun isoforms, BunA and BunF (Figure 1D). Furthermore, ubiquitous overexpression of *bunA*, but not of *bunB *or *bunC*, was sufficient to rescue the pinhead phenotype and the recessive lethality of *bun *(Figure 1C, Methods, and data not shown). The fact that neither *bunB *nor *bunC *mutations were found in our screen could be explained in two ways. Either *bunB *and *bunC *specific exons (as well as the 3' exon common to all transcripts) were not hit by the mutagenesis because they represented considerably smaller targets, or mutations in *bunB *and *bunC *did not result in a growth phenotype. In order to assess the growth function of the individual isoforms, we generated isoform-specific deletions presumably resulting in a complete loss-of-function of the respective isoform. We also generated a deletion affecting all isoforms (Figure 1E, Methods). All EMS mutations and deletions affecting both *bunA *and *bunF *are subsequently referred to as *bunA *alleles. Animals homozygous for the *bunA *deletion alleles (*A-211B *and *A-149B*) as well as for the allele affecting all isoforms (*200B*) died mostly at the larval stage. The lethality of all hetero- and homoallelic combinations was rescued by ubiquitous expression of a *bunA *transgene (data not shown). Conversely, the homozygous *bunB *and *bunC *mutants were viable, fertile, and of normal size. Functional redundancy of BunB and BunC could be excluded because ubiquitous overexpression of *bunA *was sufficient to rescue the lethality of allele *200B*, thus reflecting a *bunB *and *bunC *double mutant situation (data not shown).

**Figure 2:**
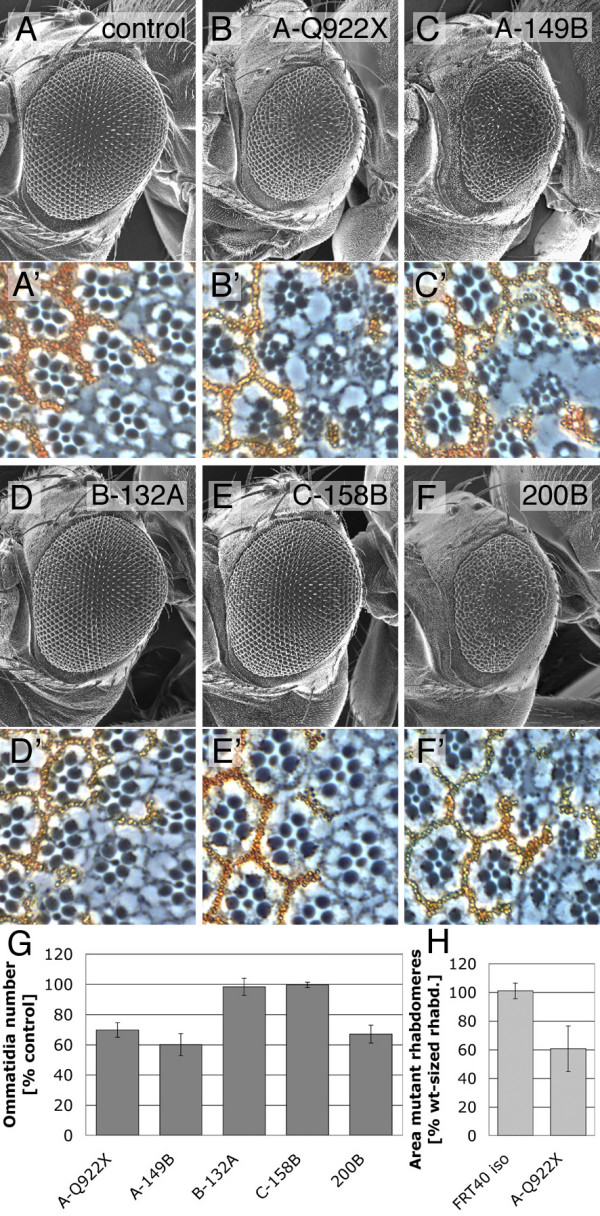
**The *bunA *growth phenotype**. (A-F) SEM pictures of mosaic eyes generated with the eyFLP/FRT system. The alleles used are indicated. A precise excision of the P-element *GE12921 *serves as control (A). Eyes largely homozygous for *bunA *mutations (B and C) and for the deletion allele *200B *(F) are small. (A'-F') Tangential sections of mosaic eyes containing homozygous mutant photoreceptors (marked by the lack of pigmentation) surrounded by heterozygous (and therefore wild-type sized) photoreceptors. A cell size reduction is apparent in clones of *bunA *mutant cells (B', C', and F'). *bunB *(D') and *bunC *(E') mutant photoreceptors do not differ from control photoreceptors. (H) Rhabdomere size is 40% decreased in *bunA *mutant ommatidia (B', only clones without differentiation defects were analyzed). The area enclosed by the rhabdomeres of photoreceptors R1-6 in unpigmented mutant ommatidia relative to neighboring pigmented ommatidia was measured (*n *= 7). Clones were induced early during development (24–48 h after egg deposition (AED)) using the hsFLP/FRT technique. (G) Statistical analysis of ommatidia number in mosaic eyes (*n *= 6) relative to control lines (*n *= 6, *FRT40A*^*iso *^is used as control for EMS-induced *bunA *alleles, and precise excisions of the respective P-element insertions for the deletion alleles). Mosaic eyes largely consisting of *bunB *and *bunC *mutant clones have a normal number of ommatidia. Eyes from female flies were examined in all analyses.

When assayed in the eyFLP/FRT system (Figure 2), the *bunA *deletion alleles and the deletion *200B *produced a pinhead phenotype (Figure 2C and 2F). The number of ommatidia in the pinhead mosaic eyes was significantly reduced compared to control flies indicating that cell number was impaired (Figure 2G). In contrast, *bunB *or *bunC *mutant mosaic eyes did not show an alteration in ommatidia number (Figure 2D and 2E). The effects on cell size were determined in tangential sections of mosaic eyes by measuring rhabdomere size (Figure 2A'–F'), revealing that homozygous *bunA *mutant photoreceptors were 40% smaller than the surrounding heterozygous (and therefore phenotypically wild-type) photoreceptor cells (Figure 2H). This cell size reduction was strictly cell-autonomous. In some clones of *bunA *mutant cells, we also observed patterning defects (see below), complicating an accurate quantification of the cell size phenotype. Whereas allele *200B *behaved very similarly to the *bunA *deletion alleles (but consistently produced milder phenotypes), *bunB *and *bunC *mutant clones displayed neither patterning defects nor a cell size reduction. Taken together, the characterization of the isoform-specific *bun *alleles revealed that *bunB *and *bunC *are dispensable and not involved in growth regulation, at least under standard culture conditions.

### Allelic series of *bunA *alleles

We attempted to further characterize the *bunA *specific growth deficit. The recessive lethal *bunA *alleles were crossed to a deletion removing the *bun *locus (Methods; and data not shown) to classify the alleles according to the strength of the hemizygous larval phenotypes. The allele affecting all Bun isoforms (*200B*) was considered to be null because Bun proteins lacking the TSC-box and the leucine zipper are likely to be non-functional. *200B *mutant larvae were massively reduced in body size, reached the third larval instar with a delay of 24 hours, and died within few days after having reached this stage. However, the *bunA *deletion alleles (*A-149B *and *A-211B*) and the *bunA *EMS alleles leading to a stop codon displayed stronger phenotypes. They developed more slowly and died during the second and third larval instars. In the case of the *bunA *EMS alleles leading to a stop codon, very few L3 larvae survived up to 14 days (control larvae pupariate after five days) and in rare cases they initiated pupariation but died as pseudo-prepupae. *A-R508W *and *A-P519L *displayed much milder phenotypes. These mutant larvae accumulated more mass, most of them developed into L3 larvae, and some into prepupae.

We concluded the following allelic series: strong *bunA *alleles (*bunA *deletion alleles > *bunA *EMS alleles resulting in a premature stop) > *200B *> *A-R508W*, *A-P519L*. The larval phenotypes of strong *bunA *deletion and EMS alleles are more severe than those displayed by the deletion allele affecting all *bun *isoforms, indicating that lacking *bunA *function alone is more deleterious than lacking all Bun isoforms. Consistently, heteroallelic combinations of strong *bunA *alleles with *200B *resulted in intermediate larval phenotypes. The balance of Bun isoforms may indeed be important because the Bun proteins share the C-terminal putative DNA-binding TSC-box and the leucine zipper for dimerization. Thus, if only *bunA *is lacking, the short isoforms may form unfavorable dimers, or they may take over the binding to common interaction partners or the regulation of common target genes.

### BunA function is required to promote cellular growth

**Figure 3:**
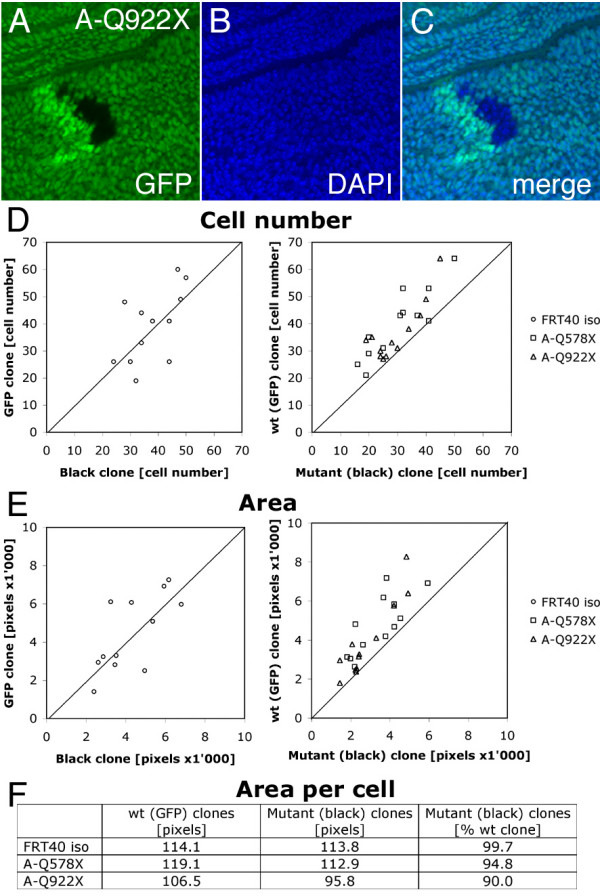
**Cell number and cell size are reduced in *bunA *mutant tissue**. (A-C) A part of a wing imaginal disc containing a twin-spot clone is shown. The clone of *bunA *homozygous mutant cells (black) and its wild-type sister clone (bright green) were induced by the FLP/FRT recombination system (genotype *y, w, hsFLP/y, w; FRT40A, Ubi-GFP/FRT40A, bun*^*A*-*Q*922*X*^, heat shock for 25 min at 34°C 24–48 h AED), and larvae were dissected 51–52 h after induction of mitotic recombination. (B) Nuclei are visualized by DAPI staining. (D-E) Statistical analyses of twin-spot clones (*n *= 12 for every genotype). Control clones (*FRT40A*^*iso*^) contain roughly the same number of cells (38 ± 7) as their sister clones (39 ± 13) and cover a comparable area (4291 ± 1506 and 4471 ± 1976 pixels, respectively; data points are evenly distributed around the straight line with the slope m = 1). However, cell number and clone area are reduced in *bunA *mutant clones (shift of data points). Homozygous mutant *A-Q578X *and *A-Q922X *clones contain significantly (*p *≤ 0.05) fewer cells (30 ± 11 and 30 ± 8, respectively) than their sister clones (40 ± 13 and 37 ± 11, respectively). The areas covered by *A-Q578X *and *A-Q922X *mutant clones (3424 ± 1256 and 2826 ± 1216 pixels, respectively) are smaller than the areas covered by their sister clones (4785 ± 1516 and 3903 ± 1939 pixels, respectively; *p *= 0.013 and *p *= 0.06). The effect on clone area is slightly more pronounced than the effect on cell number, indicating a decrease in size of *bunA *mutant cells. (F) The average area of the *bunA *mutant cells is 5% (*A-Q578X*) and 10% (*A-Q922X*) smaller than the average area of the wild-type sister cells.

In order to assess the growth behavior of cells lacking *bunA *function, we used the strong *bunA *alleles to perform a clonal analysis in larval wing discs (Figure 3). Using the *FLP/FRT *technique [40] mitotic recombination was induced early in larval development by a heat shock. Recombination events led to the generation of two adjacent clones termed twin-spot clones. In this way, clones homozygous for a *bunA *allele (marked by the absence of GFP) could be compared to adjacent wild-type sister clones (marked by strong GFP expression due to the presence of two GFP transgenes; Figure 3A). Nuclei were stained with DAPI to depict individual cells (Figure 3B). Every clone homozygous mutant for a *bunA *EMS allele (*A-Q578X *or *A-Q922X*) contained fewer cells than its corresponding wild-type sister clone (Figure 3D). Consistently, the homozygous mutant clones covered a smaller area than their sister clones (Figure 3E). The reduction in clone area was slightly more pronounced than the reduction in cell number. Hence, the area covered by a single *bunA *mutant cell was smaller than the area covered by a wild-type cell (Figure 3F).

The reduced cell number in clones of *bunA *mutant cells could be due to a decrease in cellular growth or to an increase in apoptosis. Caspase-3 is one of the key executioners of apoptosis [41] and it is activated by proteolytic cleavage [42]. Staining for cleaved Caspase-3 in proliferating larval wing discs did not reveal enhanced apoptosis in *bunA *mutant tissue (data not shown). Furthermore, blocking caspase-mediated apoptosis by the expression of either baculovirus p35 [43] or *Drosophila *inhibitor of apoptosis 1 (DIAP1) [44] did not substantially suppress the *bunA *pinhead phenotype (data not shown). Thus, the *bunA *growth phenotype is caused by an autonomous reduction in cell size and a reduction in cell number, and apoptosis does not significantly contribute to the reduced proliferation rate.

### Flies with reduced *bunA *function are growth-deficient

**Figure 4:**
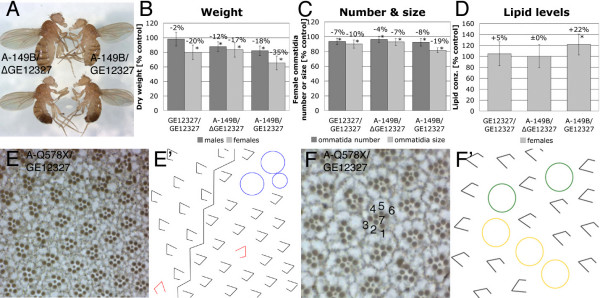
**Viable *bunA *mutant flies are small, have elevated lipid levels, and display eye differentiation defects**. The EP-element *GE12327 *inserted in the 5' UTR of *bunA *(intron of *bunF*; Figure 1E) is a hypomorphic *bunA *allele and gives rise to adult flies either homozygous or in combination with *bunA *alleles. (A) Homozygous *bunA *mutant females (top right) are smaller than heterozygous females (top left). A precise excision line of *GE12327*, termed *ΔGE12327*, serves as control. (B-D) Statistical analyses of weight, ommatidial size and number, and lipid levels of hypomorphic *bunA *mutants. All results are shown relative to values of *GE12327/ΔGE12327 *control flies (= 100%). Significant changes (*p *≤ 0.05) are marked by asterisks. The allele *A-211B *behaved akin to *A-149B *in all assays. (B) Flies with reduced *bunA *function are lighter than control flies. Allele *A-149B *affects body weight in a dominant manner. 100% corresponds to 0.370 mg in females and 0.197 mg in males, respectively; *n *≥ 35. (C) Eyes of hypomorphic *bunA *mutant females contain fewer and smaller ommatidia, indicating that both cell number and cell size are reduced. Again, allele *A-149B *dominantly lowers ommatidia number and size. 100% corresponds to 727 ommatidia; *n *= 8. (D) Females with severely lowered *bunA *function (*A-149B/GE12327*) have elevated lipid contents. 100% = 0.697 cal/mg fresh weight; *n *= 10. (E and F) Tangential eye sections of *A-Q578X/GE12327 *females reveal differentiation defects, schematically illustrated in (E' and F'). (E') The zigzag line demarcates the equator. Underrotated ommatidia are shown in red, and blue circles indicate fused ommatidia. (F') Yellow circles represent R7 to R1/6 transformations, and green circles indicate R4 to R3 transformations.

The P-element *GE12327 *inserted in the 5' UTR of *bunA *(Figure 1E) – therefore most likely affecting the *bunA *transcript – turned out to be homozygous viable and enabled us to assess the *bunA *growth phenotype in adult flies with reduced *bunA *function. Flies homozygous for *GE12327 *eclosed with a delay of about 36 hours, and 40–70% adult flies of the expected Mendelian ratio were recovered (with a slight bias towards males, 55–60%). The *bun*^*GE*12327 ^males and females were both sterile. In combination with strong *bunA *alleles, *GE12327 *caused more severe phenotypes (15–40% sterile flies eclosed with a delay of 48–60 hours, and 60–70% of them were male). These hypomorphic *bunA *mutant flies were small (16% and 34% reduction in dry weight in males and females, respectively; Figure 4A and 4B). Furthermore, a dominant effect on dry weight was observed for the alleles *A-149B *and *A-211B *in both sexes. *GE12327 *did not dominantly diminish body weight (data not shown), and only females homozygous for this hypomorphic allele were growth deficient (20% reduction in dry weight).

A quantification of ommatidia number and size in eyes of hypomorphic *bunA *mutant viable females revealed fewer and smaller ommatidia (Figure 4C). Consistently, a reduction in wing area was detected in females carrying one of the strong *bunA *alleles, *A-149B *or *A-211B*, in combination with either *ΔGE12327 *(a precise excision allele that we used as control) or *GE12327 *(data not shown). The small wing phenotype was predominantly caused by a reduced cell number since the cell density was not significantly increased (data not shown). Additionally, we found that the small *bun*^*A-149B or A-211B/GE12327 *^females contained more lipids (total triglycerides) per weight than controls (Figure 4D).

However, the defects observed in hypomorphic *bunA *mutants were not solely related to growth and metabolism. *bunA *mutant viable flies, primarily females and combinations of *GE12327 *with strong *bunA *alleles, also displayed a rough eye phenotype. Various subtle differentiation defects contributed to the rough eye, including under-rotation of ommatidia (especially around the equator, Figure 4E and 4E'), fusions of ommatidia, and cell fate transformations. With a low frequency, the R4 photoreceptor cell adopted the cell fate of the R3 cell, and a few R7 cells transformed to R1/R6 cells (Figure 4F and 4F'). Eye sections containing large *bunA *mutant clones (produced with EMS or deletion alleles) revealed the same differentiation defects (data not shown). The cell fate transformation phenotypes are similar to the eye phenotypes associated with low Notch activity [45-48], consistent with a role of *bun *in Notch signaling [49].

### A sensitized system reveals dominant negative effects of *bunB *and *bunC*

**Figure 5:**
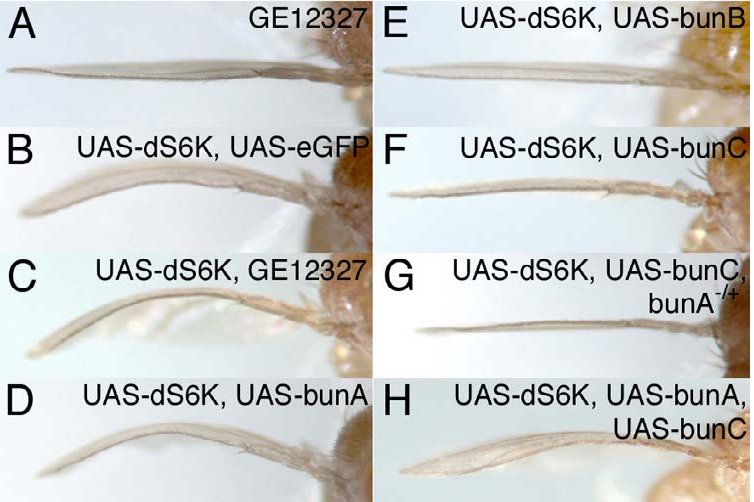
***bunB *and *bunC *can interfere with *bunA *function**. (A-H) Side view of wings overexpressing the indicated *UAS *transgenes under the control of the *apterous-Gal4 *(*ap-Gal4*) driver line. (A) EP-mediated expression of *bunA *does not produce a wing bending phenotype. (B) Overexpression of *dS6K *in the dorsal wing compartment leads to wing bending (*eGFP *was co-expressed as a control). The *dS6K *bent-down wing phenotype is enhanced when the EP-insertion *GE12327 *(C) or a *UAS-bunA *transgene (D) is used to co-overexpress *bunA*. Expression of *bunA *from the EP consistently results in stronger phenotypes than from the *UAS-bunA *transgene. (E) Co-overexpression of *bunB *leads to a complete suppression of the *dS6K *bent-down wing phenotype. (F) Expression of *bunC *suppresses the *dS6K *bent-down wing phenotype, and the suppression is even stronger when a copy of *bunA *is removed (G), indicative of a dominant negative effect of *bunC *on *bunA*. Consistently, co-expression of *bunA *interferes with the suppressing effect of *bunC *(H).

We next tested the effects of *bun *overexpression. Driving the expression of *bunA*, *bunB*, or *bunC *with various Gal4 lines did not result in any apparent growth alterations (Methods). Therefore, a sensitized system in the *Drosophila *wing was used to investigate whether overexpression of *bun *affected tissue growth. Compartment-specific expression of ribosomal protein S6 kinase (dS6K, a signaling component acting downstream of dTOR) in the dorsal compartment of the wing imaginal disc was achieved by means of an *apterous-Gal4 *(*ap-Gal4*) driver line (Figure 5B; [50]). Overexpression of *dS6K *in the dorsal compartment causes a subtle increase in cell size in the dorsal epithelium of the wing, and owing to the tight association of the dorsal and ventral wing epithelia a bending down of the wing ensues. The degree of bending is thus a sensitive measure for changes in cellular growth. Whereas *ap-Gal4 *mediated expression of *bunA *did not affect the curvature of the wings (Figure 5A and data not shown), co-overexpression of *dS6K *and *bunA *in the dorsal compartment enhanced the bent-down wing phenotype (Figure 5C and 5D). In contrast, co-expression of *bunB *suppressed the *dS6K*-mediated phenotype completely (Figure 5E), and a substantial suppression was achieved by co-expression of *bunC *(Figure 5F). Since all Bun isoforms share the C-terminal putative DNA-binding TSC-box and the leucine zipper for dimerization, it is conceivable that the short isoforms BunB and BunC can act in a dominant negative manner by either forming unfavorable dimers or by competing for interaction partners or target genes. In fact, the suppressive effect of *bunC *co-expression on the *dS6K *overexpression phenotype was enhanced by removing one copy of *bunA *(Figure 5G), whereas taking out one copy of *bunA *without *bunC *co-expression did not alter the *ap *> *dS6K *wing bending. Consistently, co-expression of *bunA *and *bunC *neutralized each other's effect on the dS6K-mediated wing phenotype (Figure 5H).

Taken together, *bunA *displayed a gain-of-function growth phenotype in a sensitized system caused by compartment-specific expression of a growth-promoting gene (*dS6K*) in the developing wing. This sensitized system additionally revealed opposite growth effects of both *bunB *and *bunC*. Because lowering the gene dosage of *bunA *did slightly enhance the *bunC *overexpression phenotype and because *bunA *and *bunC *overexpression neutralize one another, *bunC *(and possibly *bunB*) is likely to act on *bunA *in a dominant negative manner.

## Discussion

Here we show that *bunA *functions in growth control. BunA positively regulates growth by adjusting cell number and cell size during *Drosophila *development. Additionally, we found that the short Bun isoforms can act in a dominant negative way on BunA function.

The *bun *genomic locus gives rise to six different transcripts. Since each transcript has at least one distinct 5' exon, the expression of the six mRNAs is likely to be controlled by separate promoters. The distinct 5' exons result in Bun proteins with individual N-termini, except for BunF that is almost identical to BunA. All Bun isoforms have a common C-terminus comprising a conserved DNA-binding domain (TSC-box) and a leucine zipper for homo- and heterodimerization [27,51]. It is conceivable that the Bun isoforms exert different functions, since BunA, but not BunB and BunC, is involved in growth control. BunB and BunC might be (partially) redundant to other proteins, for example BunD and BunE, and hence they would only exhibit their mutant phenotypes in double mutant situations. However, our data allow us to conclude that BunA is the major Bun isoform involved in growth control because restoring *bunA *function suffices to rescue the lethality and the growth deficit associated with a deletion allele that removes the TSC-box and the leucine zipper and thus presumably represents a complete loss-of-function for all Bun isoforms.

Flies with impaired *bunA *function are small due to fewer and smaller cells. Consistently, clones of cells lacking *bunA *function remain smaller than their sister clones, and the reduction in clone area is also caused by a diminution of both cell size and cell number. Since apoptosis is not obviously enhanced in clones of *bunA *mutant cells, we conclude that *bunA *is required to adjust cellular growth. In line with our results, Wu and colleagues (manuscript submitted) found that BunA exerts similar growth effects in follicle cells and in cultured *Drosophila *S2 cells.

The *bunA *growth phenotypes are reminiscent of the phenotypes caused by an impairment of insulin signaling [52-56]. Furthermore, *bunA *also affects lipid metabolism, as has been shown for insulin signaling [53,57]. Therefore, we tested whether *bunA *would genetically interact with insulin signaling components (data not shown). However, we concluded that BunA is probably not a core component of the insulin signal transduction cascade because we did not detect a clear epistatic relationship with the lipid phosphatase PTEN. It is also unlikely that BunA acts directly in the TOR signaling branch because *bunA *mutant larvae do not display the pronounced growth deficit of the endoreplicative tissues (salivary glands, fat body) that has been observed in *dTOR *and *Rheb *mutant larvae [58,59].

BunA is clearly distinct from insulin signaling components in that it also affects pattern formation. Flies with lowered *bunA *function display various eye phenotypes reminiscent of defects associated with reduced Notch signaling activity [45-48]. Dobens and colleagues [49] have proposed a model whereby *bun *modulates Notch signaling by indirectly adjusting the amount of the Notch ligand Serrate during eggshell development. A similar relationship between *bun *and Notch signaling may account for the function of *bun *in patterning processes such as photoreceptor cell differentiation. *bun *genetically interacts with the EGF receptor and Dpp (BMP-2/-4 ortholog) signaling cascades during eye development [36] as well as during oogenesis [60]. Presently, it is unclear whether *bunA *has distinct patterning and growth functions or whether it operates at the interface between pattern formation and growth regulation by integrating various patterning signals to adjust cellular growth.

BunA influences cellular growth and proliferation yet the mechanism remains unknown. In light of the putative transcriptional regulator function of BunA, it is conceivable that bunA induces the expression of growth-promoting genes or it represses the expression of growth inhibitors. However, Treisman and colleagues [36] have reported that BunA predominantly localizes to the cytoplasm in the larval eye disc. In addition, we could not detect any nuclear signal upon expression of an N- or C-terminally GFP-tagged BunA in *Drosophila *S2 or Kc cells (data not shown). Thus, BunA might shuttle between the cytoplasm and the nucleus, and its translocation to the nucleus might be tightly regulated. Alternatively, BunA could function in the cytoplasm in a process distinct from transcriptional regulation. The identification of BunA binding partners should shed light on the subcellular environment in which BunA exerts its function.

Our study on the growth-promoting function of *bunA *in *Drosophila *may influence the perspective on the mammalian homologs of Bun, especially on TSC-22 (TSC22D1). Whereas the longer isoform of TSC-22 (TSC22D1.1) is similar to BunA (and BunF), the shorter isoform (TSC22D1.2) resembles BunB, BunD, and BunE. Data from numerous studies suggest that TSC22D1.2 acts as a tumor suppressor [4-7,10-12], which is at odds with the fact that only *bunA *is involved in growth regulation in *Drosophila*, and that BunA behaves rather opposite to a tumor suppressor. The results from our *in vivo *analysis may be of special interest in this context, since the relative balance of *bun *transcripts is important (allelic series) and overexpression of *bunC *(and also *bunB*) interferes with *bunA *function in a dominant negative manner. If this interaction is conserved in mammals, we can envision the following scenario for how the *TSC-22 *locus may be involved in tumor suppression. Whereas the long TSC-22 isoform, TSC22D1.1, positively regulates cellular growth (as does BunA), the short isoform, TSC22D1.2, inhibits growth by competing with TSC22D1.1. The antagonism between the long and the short isoforms can be achieved at several levels. An excess of the short isoform could lead to the formation of non-functional heterodimers, or the two isoforms could compete for another dimerization partner. Provided that TSC-22 functions in transcriptional regulation, the two isoforms might also contribute to differential regulation of target genes. In either case, the long TSC-22 isoform could be hyperactivated as a consequence of the loss of the short isoform. Thus, the short isoform could act as a tumor suppressor by keeping the long isoform in check. Our findings should encourage further studies in mammals that distinguish between the TSC-22 isoforms and that primarily focus on the function of the long TSC-22 protein, TSC22D1.1.

## Conclusion

In an unbiased screen for growth-regulating genes in *Drosophila*, we have isolated mutations in *bunched*, the only *Drosophila *locus that encodes proteins homologous to the mammalian TSC-22 family proteins. Our genetic analysis of *bun *revealed BunA as a positive growth regulator that adjusts cellular growth and proliferation. The short isoforms BunB and BunC are not required for normal growth, but they can interfere with BunA function in a dominant negative manner. This is the first report on the different *in vivo *functions of long and short isoforms of TSC-22 family members. In light of our findings, the analysis of the tumor suppressor function of mammalian TSC-22 requires a rigorous distinction of the long and short isoform. We propose that the long TSC-22 protein (TSC22D1.1) is a functional homolog of BunA in growth regulation, and that its function is antagonized by the short TSC-22 protein (TSC22D1.2). Thus, loss of TSC22D1.2 may result in deregulated TSC22D1.1 activity.

## Methods

### Breeding conditions and fly stocks

Flies were kept at 25°C on food described in [61]. For the genetic mosaic screen *y, w, eyFLP; FRT40A, w*^+^, *cl*^2*L*3^*/CyO, y*^+ ^[62] flies were used. Clonal analyses in the adult eyes and imaginal wing discs were carried out with *y, w, hsFLP; FRT40A, w*^+ ^[40] and *y, w, hsFLP; FRT40, Ubi-GFP *(Bloomington Drosophila Stock Center, modified) flies, respectively. Complementation tests were performed with *bun *alleles *00255 *(Bloomington Drosophila Stock Center; described in [38]), *04230*, *06903*, and *rI043 *[36]. The *bunA *pinhead phenotype was rescued by driving *UAS-bunA *[36] with *ey-Gal4 *(insertion on 3^rd ^chromosome; U. Walldorf, Medizinische Fakultät, Universität des Saarlandes, Homburg, D) recombined with *GMR-Gal4 *(insertion on 3^rd ^chromosome, unpublished). In the four independent jump-out screens the EP-elements *GE12327*, *GE14917*, *GE11969*, and *GE12921 *(GenExel Inc., commercially available) were mobilized using a *Δ2–3 *transposase strain ([63]; Bloomington Drosophila Stock Center). The resulting deletion alleles were recombined onto *FRT40A *chromosomes [40]. For allelic series *Df(2L)Exel6033 *was used (Bloomington Drosophila Stock Center). For the overexpression studies in the adult wing the following fly strains were used: *ap-Gal4 *(described in [64]); *ap-Gal4, UAS-dS6K *[50]; *UAS-bunB *[36], and *UAS-bunC *(XW and LR, manuscript submitted).

### eyFLP/FRT screen, mapping of EMS mutations, and rescue experiments

The eyFLP/FRT technique [62] was used to produce mosaic flies with eyes and head capsules largely homozygous for a randomly induced mutation. The rest of the body (including the germ line) remained heterozygous and was therefore phenotypically wild-type (screen described in [30]).

The eight EMS alleles of a complementation group on 2L were mapped using visible markers and large deletions (*Df(2L)prd1.7 *and *Df(2L)Prl *failed to complement the EMS alleles; Bloomington Drosophila Stock Center) to the cytological interval 33B2-F2. Mapping data obtained with molecular markers (P-elements and SNPs, details available upon request) further narrowed down the candidate region to 33E7-F2 and pointed to the distal border of the candidate region where 5' exons of *bun *were located.

Using the UAS/Gal4 system [65], we tested whether ubiquitous overexpression at different levels – achieved by *armadillo-Gal4*, *daughterless-Gal4*, and *actin5C-Gal4 *– of *bunA*, *bunB *or *bunC *transgenes would rescue the lethality of *bunA *alleles. Although the *bunB *and *bunC *transgenes resulted in strong protein expression (as assessed by Western blots on larval lysates), they could not rescue the lethality associated with *bunA *mutations.

### Jump-out screens and allelic series

The GenExel EP-element insertions were isogenized (*y, w; GE*^*iso*^*[w+]/CyO) *prior to mobilization achieved by crossing to *Δ2–3 *flies (*y, w; Sp/CyO; Δ2–3, Sb/TM6B*). F2 males lacking the mini-white eye marker were collected after mating, and DNA of 10 flies was pooled and amplified by PCR using primers flanking the regions of interest (primer sequences available upon request). Deletions were identified by gel electrophoresis and analyzed by sequencing. Positive pools were split up to single flies to identify the individuals carrying the deletions. Deletions *A-149B *and *A-211B*, both beginning 343 bp upstream of the *bunA *start codon, removed 2513 bp and 2038 bp of genomic DNA, respectively, including regions coding for domain 1 and 2. In alleles *B-132A *and *B-181A*, the deletions extended from 217 bp upstream to 126 bp and 20 bp downstream of the *bunB *start codon, respectively. The deletion *C-158B *started 341 bp upstream of the *bunC *start codon and eliminated the whole ORF of the first *bunC *exon (613 bp in total). *200B *removed the entire coding region and the splice acceptor site of the common *bun *exon (starting 29 bp upstream of the common *bun *exon and extending for 641 bp).

Allelic series was determined by crossing *bun *alleles (*y, w; bun*^-^*/CyO, y*^+^) to a deficiency removing the *bun *locus (*y, w; Df(2L)Exel6033/CyO, y*^+^). Animals were reared on agar plates supplemented with yeast at 25°C.

### Clonal analysis

Clones in the adult eyes were induced 24–48 hours AED by a heat shock for 1 hour at 34°C in animals of the genotype *y, w, hsFLP/y, w; FRT40A, w*^+^*/FRT40A, bun*^-^. For tangential eye sections adult fly heads were cut in half using a razor blade and shortly stored in Ringers on ice. Eyes were then fixed as described in [66]. For the clonal analysis in the larval wing discs, *y, w, hsFLP/y, w; FRT40A, Ubi-GFP/FRT40A, bun*^- ^animals were given a heat shock for 25 minutes at 34°C 24–48 hours AED. Larvae were dissected in Ringers 51–52 hours after the heat shock, and the discs were fixed in 4% paraformaldehyde (in 1 × PBS) for at least 1 hour on ice. Nuclei were stained by incubation for 30 minutes in DAPI (0.5 μg/ml in 1 × PBS) at room temperature, and wing discs were mounted in Vectashield Mounting Medium. Pictures were taken using a Leica SP2 confocal laser scanning microscope.

For the quantification of the clones, ommatidia in mosaic eyes and cell number in larval wing discs were counted, and the clone area in larval wing discs was determined using Adobe Photoshop 7.0. In tangential eye sections, the area enclosed by rhabdomeres from photoreceptor cells R1–R6 was measured in mutant ommatidia (lacking pigmentation) and in neighboring wild-type sized ommatidia (pigmented). Student's t-tests were used to test for significance.

### Analysis of adult flies

Adult flies reduced in BunA function: Freshly eclosed males and females of the genotype *y, w; bun*^*GE*12327^*/FRT40A, bun*^*A*-149*B or A*-211*B *^or *y, w; bun*^*GE*12327^*/ΔGE12327 *were kept together on fresh food for two days. For weight experiments the flies were exposed to 95°C for 5 minutes and air-dried at room temperature for 3 days. The dry weight of individual flies was assessed using a Mettler Toledo MX5 microbalance. For the analysis of adult eyes and lipid contents the flies were frozen at -20°C. Single ommatidia were counted on scanning electron micrographs, and the areas of seven adjacent ommatidia in the center of the compound eye were measured using Adobe Photoshop 7.0. Lipid levels were quantified as described in [67].

Overexpression of Bun isoforms using the UAS/Gal4 system [65]: Several ubiquitous and wing-, eye-, and fat body-specific Gal4 driver lines – namely *armadillo-Gal4*, *actin5C-Gal4*, *daughterless-Gal4*, *GMR-Gal4*, *ey-Gal4*, *MS1096-Gal4*, *C10-Gal4*, *ap-Gal4*, and *pumpless-Gal4 *– were tested with *GE12327*, *UAS-bunA*, *UAS-bunB*, and *UAS-bunC*. Neither single nor combined overexpression of the constructs led to altered growth. The combinations of *actin-Gal4 *with *UAS-bunA *or *GE12327 *were lethal. *GE12327 *led to expression of BunA but not of the short Bun isoforms (as assessed by Western blots on larval lysates).

Genotypes of adult flies with wing phenotypes: *y, w; ap-Gal4/GE12327*; *y, w; ap-Gal4, UAS-dS6K/UAS-eGFP*; *y, w; ap-Gal4, UAS-dS6K/GE12327*;* y, w; ap-Gal4, UAS-dS6K/*+; *UAS-bunA, UAS-bunB *or *UAS-bunC/*+; *y, w; ap-Gal4, UAS-dS6K/bun*^*A*-211*B*^; *UAS-bunC/*+; *y, w; ap-Gal4, UAS-dS6K/+; UAS-bunA, UAS-bunC/+*.

## Authors' contributions

SG carried out the experiments shown in this study and drafted the manuscript. SO, AS and CS carried out the eyFLP/FRT screen and isolated the *bunA *alleles. FR initiated the mapping of the *bunA *alleles. XW and LR provided the *UAS-bunC *transgenic flies and shared results prior to publication. EH was responsible for the conception and funding of this study and revised the manuscript. HS conceived and supervised this study and helped to draft the manuscript. All authors read and approved the final manuscript.
